# Extracts and Gleanings

**Published:** 1871-03

**Authors:** 


### PART II.

### Abridged Extracts and Gleanings from our Exchanges

*MULTUM IN PARVO.*

THERAPEUTIC VALUE OF CHLORIDE OF AMMONIUM.—Dr. William Cholmeley states (Transact. St. Andrews Med. Grad. Asso.) that during the last fifteen years he has been in the habit of employing this medicine in cases in which he deemed it appropriate, and among them are: 1. Some forms of neuralgia of the fifth pair, especially those occurring in women beyond twenty years of age, whose strength has been overstrained by rapid child-bearing, prolonged suckling, anxiety, want or overwork. In doses of fifteen to twenty grains, given three times a day, the pain, which is usually of a dull, aching character and intermittent, is quickly relieved, and ferruginous tonics may then be prescribed. 2. In some cases of more genuine tic-dolouroux, and in hemicrania, it is invaluable. 3. Nervous headache, such as occurs in some patients after any violent emotion or strain of the nervous system, is readily amenable to the same doses mingled with chloric ether. 4. It is serviceable also in cases of myalgia, such as affects those whose work requires long maintenance of one position. 5. In sciatica, given in the same doses, in every four or six hours. 6. In lumbago. 7. In the painful sequels of rheumatic fever, and states analogous to this affecting men who are overworked. 8. Dr. Cholmeley considers it finally to have a powerful emmenagogue influence in cases of amenorrhœa occurring in delicate and nervous girls and women, especially when this has occurred after exposure to cold and wet. In such cases it may be advantageously combined with the perchloride of iron. It is also beneficial in cases of dysmenorrhœa occurring in highly nervous or rheumatic patients, and in the various ailments that accompany the change of life in women.—*The American Practitioner.*

MANAGEMENT OF THE PERINEUM IN LABOR.—Dr. Goodel, Clinical Lecturer on Diseases of Women and Children in the University of Pennsylvania, treats of this subject historically, with reference to authorities as to clinical experience, and on scientific grounds. Some of the highest authorities on the continent and in Great Britain condemn *supporting* the perineum. Professor Taylor, of Bellevue, states that as many lacerations occur in the practice of those who support the perineum as in that of those who do not. Both experience, negative and positive, and reason unite in denying this supposed duty of the accoucheur. It may be mischievous too by impairing the vitality of the perineum, the pressure of the hand preventing the free circulation of blood; or the expulsive pains may be increased by reflex irritation at the very time when it is desirable they should be curbed, lest too rapid delivery before adequate dilatation of the vulval orifice should occur. The vast majority of natural labors require no assistance whatever, provided frequent *touching* has not taken place. Whenever it seems proper to aid nature, insert one or two fingers of the left hand into the rectum, the woman lying on her left side, with her knees well drawn up and separated by a pillow, and hook up and pull forward the sphincter ani toward the pubis. The thumb of the same hand is to be placed upon the fetal head, scrupulously avoiding all contact with the fourchette. The right hand assists the thumb in making the head hug the pubis, or in retarding its advance. After a pain it presses back the head from the perineum, and thus represses reflex uterine action; it restrains the movements of the woman; it pushes up the corrugated scalp, so that no folds shall remain beneath the sharp edge of the perineum to increase the circumference of the child's head; finally it supports the emerging head and body, causing them to describe the curve of Carus.

Where forceps are used in order to avoid rupture of the perineum, as soon as this part is well distended the instrument is removed—unless this removal requires a force which might accelerate delivery—and labor left to terminate unassisted, or the head enucleated as previously mentioned.

Incision of a rigid perineum is not necessary, except the rigidity be caused by extensive cicatrices from burns, sloughs, abscesses, etc. In case rupture occurs, introduce metallic sutures at once; use the catheter for a few days, and the rent readily closes. All greasy substances applied to the maternal passages for the purpose of promoting dilatation are mischievous.—*The American Practitioner.*

Local Use of Carbolic Acid in Pulmonary Consumption and Diptheria.—Dr. Rothe writes (*Berliner klinische Wochenschrift*) that he has obtained highly beneficial effects from inhalations of carbolic acid in cases of pulmonary tuberculosis. The following is the formula in which he uses the carbolic acid: "Crystallized carbolic acid and spirits of wine, aa 1 to 2 parts; distilled water, 5 parts; tincture of iodine, 1 part. Mix. S. 10, 15, or 20 drops to 30 parts of water. To be inhaled." The iodine is added merely to lessen the unpleasant smell of the carbolic acid. Dr. R. uses the same formula, but not by inhalation, in diptheria. He makes direct application of the solution to the diseased parts by means of a mop or hair pencil. The patient uses at the same time 10 or 15 drops of the mixture to a tumbler of water. Dr. R. gives the following as a summary of his experience of the inhalation in pulmonary phthisis: 1. "The earlier in the disease the less will be the extent of the ulcerated surface, and the more nearly will it approach in character to that of a local lesion, and the greater the prospect of a successful result. 2. The inhalations may be repeated from four to six times daily for months. They should never be employed to the exclusion of such remedies as may be called for by any of the occurring symptoms. 3. The presence of fever and dyspnœa does not forbid the use of the inhalations; but when from sudden exposure to cold there ensues a catarrhal irritation of the entire extent of bronchial mucous membrane, it would seem that but little benefit is to be expected from the inhalations. 4. Hemoptysis will with the greatest promptness be arrested by the inhalation of the chloride of iron; still it is not improbable but that the inhalation of carbolic acid will act in such cases as a styptic: we know that creosote has been long employed as such in epistaxis. 5. The pertinacity with which the smell from the fumes of carbolic acid continues to pervade the atmosphere of closed apartments renders the breathing of such atmosphere beneficial in cases of diseased lungs. It is possible that in persons predisposed to pulmonary consumption, occupying an apartment filled with a similar atmosphere, the disease may be prevented. 6. The occurrence of bad symptoms is not to become the cause of despair.—*The American Practitioner.*

Chloral in Asthmatic Bronchitis.—Dr. Caspar Morris said:—I was recently in attendance upon a lady who suffers from frequently recurring attacks of bronchitis, with asthma. The skin was hot, the frequency and difficulty of respiration very great, the rales loud and musical, and the secretion very profuse, so that the mucus could be poured from the cup in an abundant, ropy stream. My attention had been arrested by the account, recently published, of the hydrate of chloral, and as she had not been relieved by any remedy which I had previously tried, except to a slight degree by chloric ether, it occurred to me that the chloral might be of service. I ordered five grains in one fluid drachm of the syrup of lactucarium of Aubergier, to be repeated in two hours if required. The two doses afforded entire relief; and she has found great comfort since from a single dose at bedtime, a good night's rest being secured by it. I mention it as a valuable aid in the treatment of this intracticable and distressing disease.—*Transactions of the College of Physicians of Philadelphia.*

Subcutaneous Injection of Bubo.—Dr. Wertheim, attached to the syphilitic and skin department of the Rudolph Hospital, Vienna, states that he has given up all attempts at dispersing buboes by causing their absorption, and now treats them by a simple and efficacious procedure—subcutaneous injection. A solution of various substances, as morphia, camphor, sulphate of copper, &c., may be used as circumstances require, muriate of morphia (gr. iv. aquæ dr. ij.) being that which is usually preferable. The ripe abscess is punctured by means of a thick needle, or the tube of a strong Pravaz syringe; after most of the pus has been gently pressed out, the injection of eight or ten drops of the solution is practiced, the patient being taught himself to empty every three hours the fluid that may have collected. The injection is at first repeated daily, and after at longer intervals. Although not essential, it is better for the patient to keep in bed. The advantages of the method are that the pain in the abscess almost immediately ceases, and the other inflammatory symptoms steadily diminish; the thickened pus is gradually transformed into a thinner and thinner exudation, gradually decreasing in quantity, and in three or four weeks it ceases entirely, and no cicatrix remains. The secretion of pus is confined to the spot, and the surrounding induration gradually diminishes.—*Lancet.*

Treatment of Chilblains.—Mr. Fergus calls attention to the salve of sulphurous acid in the treatment of this affection. It should be applied either with a camel hair-brush, or better, by means of a spray-producer. One application by the latter method usually affects a cure. The acid should be used pure, and he finds Clark's spray-producer the best when both hands are free; Richardson's when only one is so. A good wash for hands or feet affected with chilblains is sulphurous acid three parts, glycerine one part, and water one part. The acid is particularly useful in the irritating, tormenting stage of chilblains.—*Lancet*, Nov. 2, 1870—*Practitioner*, Jan. 1871.

Miasmatic Hematuria.—Dr. John W. Peavy, of Barton, Miss., communicates the following: "I have found this very troublesome affection yield I think more rapidly under the use of nitro-muriatic acid, morphine, and quinine than to any other means. In a few cases small, but *very* small, doses of calomel may be required. I think I have seen the chlorate of potash act well, when largely diluted, as a depurator."—*American Practitioner.*

Treatment of Infantile Diarrhea.—Dr. R. W. Foss recommends the powder or mucilage of gum arabic, one part to three of water, in the diarrhea of infants. He adds a quarter of a grain of gray-powder to five grains of the powdered gum for cases in which the stools are green, or pure fluid and involuntary.—*Ibid.*

Croton Oil in Scarlatinal Dropsy.—Dr. Liddell states that in cases of dropsy following scarlet fever he has obtained highly satisfactory results from one eighth to a quarter of a drop of croton oil, repeated every two hours until free purgation is produced. He administers it daily until the dropsical symptoms subside.—*Ibid.*

Indian Hemp in Menorrhagia and Dysmenorrhea.—Dr. Silver (in the Medical Times and Gazette,) recommends twenty-five minim doses of the tincture of Indian hemp in these affections. The remedy is especially adapted to the functional variety of these diseases.—*Ibid.*

Bilious Colic.—The great point is to relieve the spasms, and prevent its occurrence; this should be accomplished as quickly as possible. After it is stopped, a dose of castor oil should be taken, so that the bowels may be cleansed. Hot fomentations may be applied to the bowels during the attack. The following remedy may be given at once, with the utmost confidence that it will immediately cure this complaint in from five to thirty minutes. Try it and you will be convinced of its specific powers in curing bilious and all colics:

| | |
|---|---|
| ℞—Gum myrrh, powdered, . . . | ℥ ss. |
| Capsicum, . . . . | ʒ iiss. |
| Caustic Potash, . . . . . | ℥ ss. |
| Camphor, . . . . | ʒ vi. |
| French Brandy, . . . . . | 1 qt. |

Mix and shake well.

*Dose*—Half to one teaspoonful in a gill of hot water, for adults. Give every thirty minutes till well. Afterward a slight cathartic should be taken to move the bowels gently, often taking some for three or four days until the parts are well. I have never known this remedy to fail in effecting a cure, and I have used it privately for many years. In conclusion, I beg the reader not to cast away the above remedy, but to rely on it with the utmost confidence that it will effect the purpose desired.—*Eclectic Medical Journal.*

HÆMORRHOIDS.—The following local application is valued highly by Dr. A. E. Hull, of New York in this affection.

℞. Manna, ℥iij. Dissolve in boiling water, q. s., so that it will be about the consistence of thick cream upon cooling; add sulphur, ℥iss, to this, previously triturated in a mortar with mercury, q. s., to give the sulphur the color of gunpowder. The manna should be mixed with the sulphur; add rhei pulvis, q. s., to make mass, and divide into balls about the size of rifle bullets. Set away to harden, after being rolled in rhei pulvis. One of the balls, dipped in olive oil, may be introduced up the rectum every other night, as the case may be.—*Drug Circular and Chemical Gazette.*

NUX VOMICA IN THE DYSPEPSIA OF HYPOCHONDRIACS.—*En Fait de Therapeutique.*—There are, as always, one or two items or suggestions worthy of being placed in the budget. Prof. Trastour, of Nantes, has occasion to highly praise the employment of nux vomica in all forms of atonic dyspepsia, and especially as a relief for the painful digestions so common among the hypocondriacs. His theory is based upon the two facts, that nux vomica stimulates and regulates the activity of the spinal cord, especially in regard to its reflex action, and that the integrity of the functions of the grand sympathetic is subordinated to the regular accomplishment of the functions of this part of the nervous system.

The following is a useful formula :—

| | | |
|---|---|---|
| ℞—Pulv. nux vom. . . . . | 1—4 | grammes. |
| Pulv. cassiæ ligneæ . . | 2 | " |
| Carb. calc. or carb. mag. . | 2 | " |

M.—ft. pulv. 20.

One powder at the beginning of each meal, in unfermented bread.

M. Trastour, like many of his confreres, prefers nux vomica to the salts of strychnine, both on account of its innocuousness and its efficacy in dyspepsias.—*Med. Record.*

NITRATE OF SILVER FOR OBSTINATE HEADACHE.—Dr. Vignard recommends nitrate of silver, in large doses, as an efficient treatment for obstinate headache.

He describes the case of a female who had suffered under this painful affection for more than a month, and who had been unsuccessfully treated with opiates, but who recovered perfectly after a few doses of this remedy. He believes it to be most useful in anæmic cases. It was prescribed as follows: Nitrate of silver, one grain and a half; bread, *quant. suff.* Divide into six pills—one to be taken every hour.—*Ed. Med. Journ.*

*Treatment of Uterine Catarrh by Internal Application of carbolic Acid.*—By W. Playfair, M. D., Physician to King's College Hospital. In a large proportion of old standing cases of uterine catarrh it is hopeless to effect a permanent cure by any means which do not act directly on the seat of the disease, which is the lining membrane of the cavity of the uterus and cervical canal beyond the external os, accompanied, of course, with secondary morbid states of the body of the uterus and cervix, such as hypertrophy, congestion, &c. Rest, applications to the exterior of the cervix, and general treatment will unquestionably cause a temporary improvement, but on a recurrence to the old habits of life all the old symptoms return. There are serious objections to intra-uterine injections, unless the os is first dilated with laminaria tents, as they are apt to bring on severe uterine colics. By means of fine probes of whalebone or flexible metal, round which a thin film of fine cotton wool is wrapped, alterative applications can readily be made to the interior of the uterus, without pain or danger. In the very numerous cases in which this plan of treatment has been carried out, in no single instance has anything but the greatest benefit accrued. It is no doubt advisable to select the cases judiciously, and where there is much uterine tenderness, intra-uterine treatment should be postponed until this has been diminished by rest, leeching, &c.; but with proper precautions, the treatment is perfectly safe. A concentrated solution of carbolic acid, eighty parts to twenty of water, is used; and it acts so well that for a long time nothing else has been employed. After the first application the discharge is sometimos increased, but after the second or third it is generally greatly diminished, and a single application is often sufficient to cure superficial erosions of the cervix. As a rule, there is no difficulty in passing the probes, as in true uterine catarrh the os is invariably patulous. As the case improves the patulous state of the os diminishes, and this is found to be one of the most certain signs of improvement.—*Medical News and Library.*

Belladonna has been highly recommended in incontinence of urine, and especially in that form which proves so troublesome to children. The powder or extract administered in small doses, two or three times a day, for a few weeks, will often be found entirely successful. In nervous cough, spasmodic dyspnœa and hiccup, this remedy is often found servicable.

SAMBUCUS CANADENSIS IN ALBUMINURIA.—A number of cases given by Dr. R. Macnut, of Marshall, Mo., in the Am. Jour. of Med. Sciences, show conclusively the therapeutic value of this remedy in albuminuria. The inner bark of the common elder is to be steeped in hard cider, and used in ounce doses, three or four times daily. The improvement in a few days was quite marked, and recovery in all the cases has been complete.—*Am. Jour. of Med. Sciences.*

DR. METTAUER'S Aperient Solution, which he highly prizes, is as follows :

| | |
|---|---|
| ℞ —Aloes Socat, . . . | ℥ iiss. |
| Super-Carb. Sodæ, | ℥ vi. |
| Water, . . . | Oiv. |
| Spirit Lavend. Comp. . . | f. ℥ ii. |

Digest 14 days and filter. Age improves the quality and taste. It is used in weak digestion, with costive habits, dyspepsia, &c. He says all who try it, never fail to like it. We know it to be good—and if we did not, from the character of Dr. Mettauer, we could recommend it.

THE TINCT. OF GELSEMINUM SEMPERVIRENS IN INCONTINENCE OF URINE.—Some have used it with great success in doses of from five to ten drops, three or four times a day, when belladonna and other remedies had failed.

NITRATE OF SILVER IN THE VOMITING OF PREGNANCY.—In many cases the following pill is found very useful : Arg. nit. gr iv opii gr. viii. in pill xvi. Give one pill per day.

PRESCRIPTION FOR OPHTHALMIA GRANULAR LIDS.—℞. Tannin 5 parts, distilled water, 20. Dissolve and add ten of gum arabic, and strain. This is a most valuable application in granular lids and other affections of the eye. In chronic varicose opthalmia, one to three drops of creosote to one ounce of water, makes a valuable collyrium, dropped into the eye several times daily.

ARSENIC IN UTERINE AFFECTIONS.—Dr. Lacock thinks highly of arsenic in this class of diseases. In menorrhagia, leucorrhœa, uterine hemorrhage, threatened abortion, and after delivery, Dr. A. Burns speaks of arsenic as a most reliable remedy. In leucorrhœa, he gives five drops of Fowler's solution, three times a day, until a cure is effected.—*Med. Gazette.*

POTASSÆ CHLORAS.—At the Rudrep Hospital, Vienna, this remedy has been used by enema in cases of dysentery, with excellent results ; blood ceases to appear in the dejections after the first clysta. They used potass. chloras, ℈i. ad. Aquæ distil. ℥ ij.

WHOOPING-COUGH TREATED BY CHORAL.—Dr. A. Ferrand, after failing with other remedies, has used choral successfully in whooping-cough, which he gave in syrup in the proportion of 2 grammes to 150 of syrup. The first three days he gave 2 spoonsful—each spoonful having 25 centigrammes of choral—each evening. After this, three spoonfuls were prescribed and regularly given. Recovery was speedy.—*Boston Med. and Surg. Journal.*

BURNS AND SCALDS.—Dr. S. B. Judkin dissolves white lead in flax-seed oil, to the consistency of milk, and applies over the entire burn or scald every five minutes. It gives relief soon and permanently.—*Jour. Materia Medica.*

BROMIDE OF POTASSIUM IN DYSMENORRHŒA.—The following prescription has given good results in dysmenorrhæa:

℞—Bromid. potass. . . . . ʒ ii.

Aquæ pure . . . f. ℥ ij. M.

S. A teaspoonful in water, an hour after each meal.

The patient should commence its use two or three days before the expected time, and continue it until the amount prescribed above is used—repeating the same at each subsequent period so long as it may be needed.

TREATMENT SYCOSIS.—Mr. Stewart employs successfully, in every case of sycosis, a simple solution of nitrate of potash. A salurated, watery solution, should be applied, three or four times daily over the pustules and the whole diseased surface. If the pain caused by the application is too great, the strength of the solution is to be reduced until it can be tolerated. Another agent of ancient use, but lately revived, is turpentine. We have used it with striking success in several cases recently.—*Med. and Surgical Reporter.*

SKIN DISEASES.—The following formula, as an application in skin diseases, attended with little discharge, will be found satisfactory:

℞—Ferri pulv., . . . . ℥ i.

Cinchonæ rubræ, pulv. . . . ℥ ss.

Boracis pulv. . . . . ʒ ij.

ol. Morrhuæ, q. s., for ungent. M.

A combination which forms a coating as impervious to air as collodion, and which the writer has employed with happy results in several cases of eczema, is prepared by adding ʒ ij., quin. sulph. to ℥ ss. aa tinct. Ferri chlo. and Tinct. Cinchonæ—the parts to be painted with two or three coats.—*Med. and Surgical Reporter.*

Coal Oil in Phthisis.—Dr. C. H. Wells has used coal-oil with good results in Phthisis, in teaspoon to dessert spoonfuls, three times a day. Another gentleman has found advantage from the following combination :

℞—Tinct. cimicifugæ . . . f. ℥ v.

Olei Fusil, . . . f. ʒ i.

Spts. Frument, . . . f. ℥ xv.

Sig.—f. ℥ ss, thrice daily.

[*Med. and Surgical Reporter.*

Intermittent Fever treaeed by the Iodide of Potassium. Dr. S. L. Abbott reports a case of intermittent fever successfully treated by the iodide of potassium after failing with sulph. quinine. He used the following formula :

℞—Potass. iodidi, . . . . gr. v.

Fl. ext. quassiæ, . . . .f. ʒ ss. M.

S.—This quantity before each meal.—*Med. and Surg. Rep.*

Dyspepsia from Fermentation of the Ingesta.—In this form of dyspepsia, the sulphite of sodæ in 5 grain doses, or carbolic acid in 1 grain doses, will prove beneficial.

Uses of Carbolic Acid.—Dr. C. F. J. Lehlback uses carbolic acid in solution of 5 to 20 grains to the ounce of glycerine and water, as a dressing to wounds. The benefit was marked in arresting suppuration and exciting rapid granulation. In *carbuncles* he uses *carbolized poultices*, and after a forced or spontaneous opening of the carbuncle, a solution of carbolic acid in glycerine —ʒ ss to ʒ iss to the ounce. In conjunctevitis, he thinks it is valuable. In burns and scalds, he esteems it of great value, ʒ i to ℥ vi of glycerine. In minor burns and scalds, one application of the ordinary crude fluid article will almost invariably arrest the pain in a few moments and prevent subsequent vesication. We have used carbolic acid solution with great advantage as an injection in the treatment of buboes.

Lumbago and Sciatica.—Dr. D. J. Gish esteems the following a " superior remedy " in lumbago and sciatica :

℞—Tinct. Xanthoxylum berries, . ℥ iv.

Wine Tinct. Colchicum sem. . ℥ ii.

Spirits nit. dulcis, . ʒ ii.

Ol. terebinthinæ, . ʒ ii. M.

S. A teaspoonful diluted in a half glassful of water. He also gives it in chronic rheumatism of back and lower extremities—three times a day after each meal. Only use it in non-inflammatory rheumatic affections.

Improved Iodine Formula.—Dr. F. P. Mann, of Brooklyn, has employed successfully the following formula, where ordinary doses of iodine and iodide of potassium have failed:

℞—Iodide, . . . . . . gr. xvj.

Iod. Potassium, . . . . gr. xxxii.

Aquæ puræ, . . . . ℥ ij.

Syr. empyreumat, . . . ℥ vi.

Ess. gaultheriæ, . . . . ʒ ii. M.

S. A table-spoonful, three times a day, one hour before meals. The solution should stand 24 hours before using.

Sulphurous Acid in Syphilitic Ulceration of the Throat.—This acid, in the form of spray, has been found highly beneficial, used three times a day, to syphilitic throat ulcerations—a cure being effected in three weeks.—*British Med. Jour.*

In Common Catarrh.—Dr. Scudder uses the following:

℞—Tinct. Verat.. . . . . . ʒ ss

Tinct. gelsemini, . . . . ʒ i.

Aquæ, . . . . . ℥ iv. M.

S. A teaspoonful every one to two hours.

Dyspepsia.—J. W. Craigin, M. D., (*Med. and Surg. Reporter*) says: "If the patient is troubled with supra-obital headache, constipation, cold feet, and clay-colored stools, I would advise the compound rhubarb pill, for constipation; for featus, the following:

℞—Acidi Carbolic, . . . . gr. i to ii.
Glycerine, . . . . ℥ i. M.

S.—Teaspoonful three times a day.

℞—Elixir Pepsine, Strych et. Bismuth, q. s.

S. Teaspoonful directly after meals. I have found the elixir to be of great benefit in dyspepsia. I believe it will cure when nothing else will."

Aconite in Lichen and Prurigo.—The following has been used with much advantage:

℞—Ext. Aconiti, . . . .
Ext. Taraxac, aa, . . . . grs. xv. M.
ft. pill No. 40.

S.—One or two be taken, morning and evening.

Chronic Rheumatism.—

℞—Syrp. Sarsaparilla, . . . . ℥ iii.

Tinct. Colchici, . . . . ℥ i.

Iodidi Potassæ, . . . . ʒ iii. M.

S.—Thirty drops to be taken three times a day.

NEURALGIA PILL.—The following has been found very effectual in the relief of neuralgic affections:

℞—Zinci Cyanidi, . . . gr. vi.<br>

Quiniæ Sulph. . . . gr. ix.<br>

Morphiæ Sulph. . . . gr. iss.<br>

Ext. Belladonnæ, . . . gr. iii. M.<br>

ft. pil. No. vi.<br>

S.—One pill, every six hours until relieved.

SYPHILITIC PNEUMONIA—or an inflammatory consolidation of the Lungs, which owes its origin to the poison of syphilis, is becoming much more common than of former years. It is not unfrequently we meet with cases presenting the physical signs of pneumonia, and some of the evidences of phthisis, evidently depending upon constitutional syphilis, and readily yielding to the proper treatment for syphilis.

FOR SLIGHT UTERINE HEMORRHAGE.—In feeble constitutions, we know of no remedy more simple or preferable, than sulphuric acid, prepared as follows:

℞—Acid sulphuric, . . . f. ℥ iss.<br>
Aquæ dist. . . . . . ℥ xivss.<br>
Mix gradually. Dose from 10 to 30 drops, in a little water, three times a day. It is also one of the best remedies for the arrest of profuse perspiration, especially as connected with phthisis pulmonalis.

LUPULIN AS A SEDATIVE.—It has been very successfully used to allay the morbid erethism following gonorrhœa, and to prevent chordee. It is said to answer this purpose much better than camphor, which often irritates the digestive organs, and fails to produce the desired effects. It also contains a bitter element which acts as an admirable tonic—particularly with strumous patients—improving the appetite and strengthening the digestive functions. It is worthy of trial.

DIFFICULT MICTURITION IN WOMEN.—In the irritable bladder, which is sometimes called "gravel," in women, the following will often "act like a charm":

℞—Creasote, . . . . . gtt. xij.<br>

Vinegar—a few drops.<br>

Cinnamon water, . . . ℥ iv. M.<br>

S.—Table spoonful four to six times a day.

CREOSOTE IN DYSENTERY.—In cases attended by great irritation, and by fœtor of the evacuations, we have frequently witnessed the best possible effects from the use of Creosote. It should be given in solution of gum arabic mucilage or glycerine in the proportion of twenty drops to four ounces. Dose one teaspoonful every three to six hours, for an adult, according to circumstances.

Carbolic Acid in Diptheria.—The following has been successfully employed :

℞—Acidi Carbolici, . . . gtt. xxv.

" Acetici, . . . f. ʒ ss.

Glycerine, . . . f. ʒ ii.

Aquæ dist. . . . f. ℥ vi. M.

S. Used as a lotion. It is also recommended in putrid sore throat, and in sloughing and phagedenic ulcers.

Chronic Gonorrhœa.—Glycerine of Tannin.—The following injection is of great service in cases of gleet and chronic gonorrhœa :

℞—Glycerine of Tannin, . . ℥ iii.

Olive Oil and Mucilage, aa . ℥ i. M.

If used in the acute stage, it should be diluted with equal parts of mucilage.

Glycerine in Phthisis.—In the early stage of Phthisis the following is highly recommended :

℞—Glycerine, . . . . . ℥ ii.

Iodid. Potassium, . . . . ʒ i.

Sulph. Morphiæ, . . . . grs. ii. M.

S.—A teaspoonful before each meal, and at bed time. In the advanced stages, the following is preferred :

℞—Glycerine, . . . . ℥ ii.
Syrp. iodidi iron, . . . . ℥ ss.
Sulph. Morphiæ, . . . . grs. ii. M.

S. Teaspoonful every four or six hours.

Glycerine will be found to be the best solvent, when prescribing medicines in liquid form. It should always be used in place of syrup, when medicines are to be given in this way, to children. It never ferments. The best article should be used.

Prescription for Chronic Bronchits.—

℞—Ammoniaci, . . . . . ʒ i.

Pulv. Ipecac, . . . . . gr. x.

Mur. Morphiæ, . . . . . gr. iv.

Carb. Ammoniæ, . . . . . ʒ ii.

Mucilaginis acaciæ . . . . q. s.

Misce et divide in pilulas, . . xxx.

Sig.—Give one pill, three times a day, in cases attended with viscid bronchial secretion.

PROF. W. H. VAN BUREN'S PILL FOR CONSTIPATION.—

℞—Extracti aloes, . . . . ʒ ss.

Extracti nuci vomicæ, . . . gr. vi.

Extracti hyosciami, . . . ℈i.

Pulv. Ipecuanhæ, . . . gr. l. m.

Make pills xx. S.—One to be taken at night. This pill is of especial value in the constipation of females.—*Nep. Ther.*

A GOOD PRESCRIPTION FOR CHRONIC HEPATITIS.—

℞—Podophyllin, . . . gr. xv.

Capsici, . . . gr. xx.

Pulveris Rhei, . . . gr. xxx.

Hyd. chlo. mitis, . . . gr. v. m.

Make pills 30. S.—Give one on alternate night.

PRESCRIPTION FOR PRURITUS.—It is said that no remedy is comparable with this acid, in pruritus of the genital organs of both sexes, prepared thus :

℞—Alcohol, . . . ℥ vi.

Acid Hydrocyan . . ʒ iss. m.

S.—Apply to the parts twice or thrice a day. We know this to be good in this very annoying and troublesome affection. Dr. Thompson says that no expedient is so salutary for the intolerable itching of erysipelatous and erythmatous eruptions, as a solution of prussic acid, which may be made by adding one or two drachms of the acid to a pint of water. Another lotion used by Dr. Thompson is made in the following manner :

℞—Acid Hydrocyan, . . . . ʒ ii.

Acet plumbi, . . . . gr. xvi.

Alcohol, . . . . ℥ ss.

Aquæ dist. . . . . ℥ viii. m.

S.—To be applied three to four times a day.

NOTE ON ABORTION.—If, after a careful examination, we find the cervix preserves its normal length, figure and thickness, we must try to prevent the abortion ; but if the cervix is found shortened or distended, and the organ is assuming the ovi-form shape, we must assist by all safe means, the expulsive action. In making the examination, do not introduce the finger into the os.—*Braithwaite.*

SOLUTION FOR OPTHALMOSCOPIC INVESTIGATION.—

℞—Sulphate of Atropine, . . . gr. ½.

Aquæ dist, . . . ℥ i. M.

When it is used to dilate the pupil, a few drops will suffice, and the unpleasant effects of a stronger solution will be avoided. —*Ibid.*

Belladonna Solution.—

℞—Ext. Belladonna, . . . . ʒ i.

Glycerine, . . . . ℥ i.

Misce well. Glycerine is a better solvent for belladonna than water, for the purpose of applying around the brow, to produce dilation of the pupil. It prevents the extract from forming a hard crust, and insures with more certainty its absorption. This is also a good form for applying belladonna to the breast for the purpose of arresting the secretion of milk. We usually add half to one dram of pulverized camphor.

*Hypodermic Injection of Morphine in Neuralgia.*—This mode of treatment is best adapted to those cases where the pain is of a purely neuralgic character. It is of little or no use in cases where the pain is produced by active inflammation of the neuralemma nerve itself, or caries of bone.

*Iodine Injections in Leucorrhœa.*—In cases of obstinate vaginal leucorrhœa, when the *cervix uteri* is not involved, the following method of employing iodine injections will be found to be serviceable :

℞—Iodini, . . . . gr. i.
Potass iodidi, . . . . gr. ii.
Aqua pluvialis, . . . . . ℥ i. M

S.—To be thrown up the vagina, twice daily, and retained several minutes—the parts being previously well syringed with warm water and castile soap, and the patient placed in a horizontal position, with the hips elevated. As the solution ceases to create any sensation of warmth or excitement in the parts, it is to be gradually increased to treble its strength. The muriated tincture of iron, in the proportion of twenty drops, three times daily, will, in most cases, assist in the cure.

*Extract Cannabis in Gonorrhœa.*—Dr. M D. Mooney says he has used the following prescription in Gonorrhœa, with great success :

℞—Sugar of Milk, . . . . . ʒ ss.

Ext. Indian Cannabis, . . . gr. xx.

Mix well together, and divide into 60 powders—one to be taken every three to four hours.

*Remedy for Sore Nipples.*—It is said that three or four applications of the following compound frequently cures this troublesome complaint :

℞—Cocoa butter, . . . . . ʒ iiss.

Ext. Rhatany, . . . . . gr. xv. m.

S—Apply to the nipples.

DETECTION OF STRCHNIA IN MEDICO-FORENSIC ANALYSIS.—Dr. Weyrich relates in the *Moniteur Scientifique* a case of poisoning with strychnia, of a person accustomed to consume opium, and to whom had been given large doses of ipecacuanha, while, moreover, a portion of the contents of the intestines had to be tested for mineral poisons. The real bearing, therefore, of this case, turns upon the detection of strychnia in the presence of emetine and morphia. The strychnia was detected in an alcoholic extract of the materials taken from the corpse, by means of the reaction produced by strong sulphuric acid and bichromate of potassa, which at first oxidizes only the emetine, and this having been removed, produces the well-known purple coloration, due to the action of the bichromate and sulphuric acid upon strychnia. The morphia was detected in a separately made amyl-alcoholic solution, by means of molybdate of soda in dissolved concentrated sulphuric acid.—*Med. and Surg. Reporter.*

*Gargle for Ulceration of the Tonsils.*—

℞—Zinci Suiphus.

Chlorate Potash, aa, . . . ʒ ii.

Sage Tea, . . . ℥ viii. m.

S.—Gargle the throat frequently. This we know to be an excellent wash, in ulcerations of the tonsils, or apthous affections of the mouth.

*An Excellent Dinner Pill.*—

℞—Pulv. Capsicum, . . . . ʒ i.

Pulv. Rhei, . . . . ʒ iss.

Ext. Gentian, . . . . ʒ i. m.

Make pills 60. S.—Take two or three every day before dinner.

*Laxative and Alterative Mixture.*—

℞—Tinct. Aloes comp'd. . . . ℥ ii.

" Gentian . . . ℥ i.

" Rhei, . . . ℥ i.

Ext. Colocynth, . . . ʒ i.

" Taraxicum, . . . ʒ ii.

Bi. chlo. hydrarg . . . gr. iii.

Syrup Sarsap. . . . ℥ iii.

Aq. dist. . . . ℥ x.

Mix thoroughly in a mortar.

This is an excellent alterative for costiveness, especially when complicated with biliary derangements. Dose teaspoonful three times a day, before or after meals, one hour.

PERSISTENT HICCOUGH.—Hypodermic injection of morphia, Dr. J. Constable states, will relieve hiccough instantly, when occurring during the progress of pneumonia. It should be tried in cases arising from other causes.—*Lancet.*

